# Digital Transformation for Improving Child Health in South Asia: Do We Need a Reboot?

**DOI:** 10.2196/73053

**Published:** 2025-10-17

**Authors:** Vikas Dwivedi, Meredith Dyson, Karin Kallander, Gunter Boussery

**Affiliations:** 1 UNICEF Regional Office of South Asia New Delhi India; 2 UNICEF New York, NY United States; 3 UNICEF Pakistan Islamabad Pakistan

**Keywords:** digital health, child health, digital interventions, climate, noncommunicable disease, artificial intelligence

## Abstract

Emerging challenges, such as climate change and noncommunicable diseases, threaten the “survive and thrive” agenda for children and adolescents. These challenges have added to the existing burden of newborn and child morbidity and mortality. Digital solutions hold promising potential to address children’s evolving health needs, especially in reaching remote areas, increasing inclusion, and ensuring equitable primary health care. This commentary raises the question, are we ready to use digital solutions and artificial intelligence to achieve transformations in child health in South Asia? If not, what is the paradigm shift required to design and implement digital and artificial intelligence solutions at-scale that are effective, sustainable, and beyond small pilots?

## Introduction

Revolutionized information and communications technologies have fundamentally shifted how individuals and communities engage with their own health. Technologies allow us unprecedented access to information and empower us to take increasing ownership of our own health status and health outcomes. The World Health Organization and United Nations Children's Fund (UNICEF) Operational Framework for Primary Health Care: Transforming Vision Into Action [1] calls for health systems to “use digital technologies for health in ways that facilitate access to care and service delivery, improve effectiveness and efficiency, and promote accountability.” Digitized primary health care systems, including at the community level, contribute to enhancing all 3 pillars of the primary health care framework: improved service delivery, empowered people and communities, and multisectoral action. The potential is huge: the digitalization of health services can directly enhance quality-of-care, reduce human error, improve patient outcomes, increase efficiency [2], and lead to more equitable coverage and lower overall costs [3]. Moreover, timely, high-quality data expose inequities in health access and outcomes and enable decision-makers to identify unmet needs, including how and where to invest in health facilities, health workers, vaccines, and other supplies.

However, even as the range of digital and artificial intelligence (AI) tools available to the health sector are rapidly expanding, the impact of these for improving child and adolescent health at-scale has yet to be realized. Despite their potential, digital health interventions remain largely at the project or program level or in pilot phase due to various technical and nontechnical challenges (Textbox 1) in the development of the digital public health infrastructure required to optimize data for health outcomes [4]. Coherent data architectures are essential to build machine learning and AI capabilities that enable client-centered, quality, and efficient care across the life course and improve the continuum of care. These are still missing or weak in many countries. Data architecture coherence includes capabilities for integrated or interoperable, standardized, and automated data processing within the organization, which is crucial to frictionless integration of large-scale data streams across different locations and business functions.

**Figure 1:**
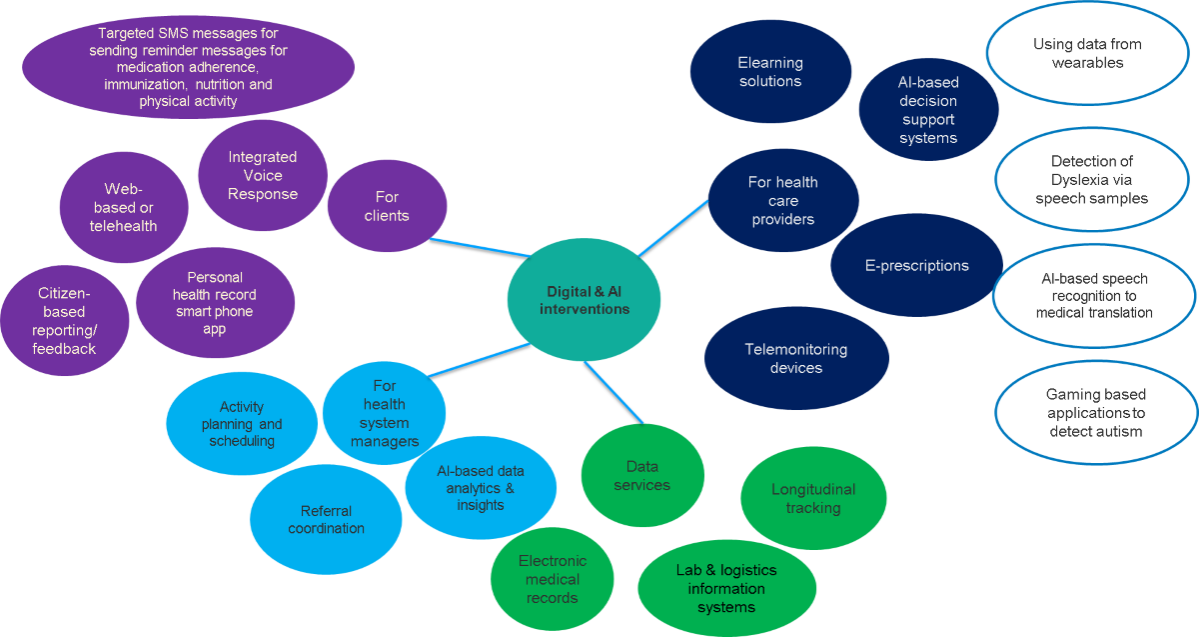
Examples of digital interventions to improve health care for children and adolescents.

The World Health Organization classification groups digital interventions into 4 categories, namely, clients, health care workers, managers, and data services. Figure 1 shows some illustrative digital interventions [4] grouped by the 4 categories.

Further, AI collects a large amount of data, often without users being fully aware or understanding the implications. This can inadvertently expose children to harmful or age-inappropriate content [5]. AI-based algorithms learn what content kids engage with, filling their feeds with it—even if it could be harmful to them or people around them [6].

**Challenges in scaling up promising digital interventions for child and adolescent health.**

Technical:

- Fragmented and unsustainable systems.
- Lack of clear standards.
- Unreliability of available data.
- Infrastructure gaps.
- Workforce capacity gaps.

Nontechnical:

- Ethics.
- Policy and governance.
- Health equity.
- Resource gaps.
- Quality of evidence.

## The Context of South Asia

Across South Asia (Afghanistan, Bangladesh, Bhutan, India, Maldives, Nepal, Pakistan and Sri Lanka), millions of children, adolescents, and caregivers lack access to effective primary health care. Each year, there are 35 million births in the region. In 2022, South Asia accounted for 34% of global neonatal deaths; every minute, 2 newborns die, and an estimated 1 in 29 children die before their fifth birthday—the second largest burden globally after sub-Saharan Africa [7]. Despite progress, an estimated 3 million out of 34 million surviving infants in South Asia did not receive the 3 recommended doses of diphtheria, tetanus, and pertussis vaccine and are not fully vaccinated. Even within countries, disparities exist which require targeted interventions to vaccinate children [8]. The region is home to the only 2 remaining polio endemic countries in the world [9].

The region also carries an alarming digital divide between boys and girls. Adolescent boys are one and a half times more likely to own a mobile phone and nearly twice as likely to own a smartphone than adolescent girls. The rate of internet usage among boys is double that of girls in Nepal and quadruple that of girls in Pakistan. Such glaring discrepancy is depriving women and adolescent girls of opportunities to engage in the digital sphere. The societal expectation about gender roles as well as online safety concerns contribute to curbing their access to the digital sphere [10]. As such, digital infrastructure access and digital literacy are becoming significant social determinants of health outcomes.

Population health and health systems in South Asia face increasingly frequent and severe threats [11]. In particular, climate change and environmental degradation are escalating threats to children’s well-being, with over 659 million children impacted in South Asia. These challenges jeopardize decades of progress in child survival, development, and protection. Responding to these threats requires strong health system readiness to communicate quickly and accurately with populations and deliver services, functions which can be significantly enhanced through digital solutions that are implemented at scale.

## Challenges With Digital Transformation and Using AI and Machine Learning to Improve Child and Adolescent Health at the National Level in South Asia

In South Asia, substantial progress has been made in introducing digital health solutions, however significant barriers to scaling them up to the systems level persist, impeding the effective utilization of digital health technologies and restricting access to primary health care for every child. Most countries in the region have not adopted standards, national architectures, or health information exchanges to enable individuals’ health information to move seamlessly through the health system [12]. This has resulted in poor coordination across the continuum of care for child health.

Machine learning and AI models increasingly use longitudinal data. However, the heterogeneity in reporting methodology and results, and the lack of electronic health records datasets and code sharing, complicate the possibility of replication [13]. Global and regional evidence shows that poor integration and coordination between applications leads to inaccurate data, negatively impacting platform effectiveness and programmatic interventions [14,15].

Further, the use of AI and big data in public health raises privacy concerns. Linkage of multiple anonymized data sources is often performed to increase the richness of data prior to analysis with AI techniques; however, this also increases the risk of reidentification of individuals or stigmatization of small groups [16]. These risks are particularly alarming when the concerned data are related to children and adolescents, with implications that could follow them throughout their lifetime.

## Reboot: How Can We Design Robust Digitalized Primary Health Care Systems for Children in South Asia?

Digital transformation is an opportunity to transform primary care and support health systems to be responsive to emerging challenges, such as noncommunicable diseases, mental health, and climate change. Transforming primary health care can help achieve the quadruple aim [17] of better care, better health outcomes, better provider experiences, and better value for money.

Integrating clinical support tools and referral systems into primary health care can help care coordination and ensure its continuity across primary, secondary, acute, and aged care services [18]. Electronic health records capture information about an individual’s health, medical conditions, medications, and key events, which can be shared for referrals and timely clinical decision-making. Digital technologies can help improve the patient’s journey. They can prevent duplication of care processes and enhance communication between providers as well as avoid unplanned hospitalizations and visits for urgent care, ensuring that people have access to timely, expert advice by telephone or telemedicine tools in health emergencies.

In national health systems, mainstreaming the use of digital tools to advance integrated primary health care across the life course and levels of care from community to specialty services is needed. Scaling up digital, AI, and machine learning tools to optimize care and close the health equity gap for all children will require a systematic approach to ensure safe, ethical, rights-based implementation and meaningful impact.

Achieving impact for children requires a paradigm shift in the way governments and partners approach digital health, with the following key considerations:

1. An urgent need to design at-scale and shift from a project-specific approach to health systems approach: Countries should develop and commit to national coherent digital and data architectures that facilitate interoperability and information exchange. Partners in the health sector need to collaborate in setting up common digital public infrastructures as essential components of the architecture and contribute to national plans rather than be siloed projects. Only when we have coherent digital and data architecture in place at the national level, will we be able to apply AI-enabled solutions that will benefit every child.
2. Strengthen governance mechanisms: Strong governance mechanisms ensure that governments are in the driving seat with support from donors and partners. This requires clear policy and regulations, structures to oversee and coordinate the design, implementation, and processes that are common for all implementers, partners, and stakeholders.
3. Leverage digital technology or AI as a platform to accelerate integration of programming and longitudinal data systems: Primary health care calls for integration of health services and a life course approach to providing a comprehensive package of health services. This can be done following a “client journey” approach and making digital tools interoperable [19]. This will facilitate provision of continuity of care critical for immunizations, nutrition, mental health, noncommunicable diseases, and other interventions.
4. Capacity and digital skills are essential for a globally competitive workforce and digitally literate citizens [20]: On one hand, efforts should be made to design tools that are easier for use by the health workforce, and on the other, adequate plans should be in place for enhancing the skills of health workers to use the solutions. Investments should be made in educating children and parents about opportunities and risks so they can navigate the digital world safely and responsibly.
5. Foster collaboration between academia, industry, government, and civil society: Collaboration across the different groups will help in coordinated efforts, joint learnings, and improving primary health care services. All of these stakeholders are critical to a robust systems approach to scaling up digital solutions for child health.
6. Establish robust data and AI governance practices to ensure child and adolescent protection and patient rights: Clearly define data ownership and access principles. A review of national AI strategies [21] concluded that current strategies lack meaningful engagement with children’s issues and identified opportunities to incorporate children’s perspectives in AI policies, suggesting human-centered principles and ethical frameworks as starting points. We must also address legal and regulatory challenges that hinder responsible data use and undermine children’s and patients’ rights [22]. The UN Secretary-General's high-level advisory body on AI​ recommends [23] the creation of a global AI data framework, developed through a process initiated by a relevant agency, such as the United Nations Commission on International Trade Law, and informed by the work of other international organizations.

## Conclusions

In the final stretch toward achieving the Sustainable Development Goals in the next 6 years, many countries in South Asia are nearing or exceeding their targets for maternal, newborn, and child mortality. New approaches are needed to pursue dual agendas of “survive” and “thrive,” and to build resilient, responsive, and adaptive health systems that are prepared to anticipate, detect, and respond to emerging threats. Reaching the last mile requires more targeted, individualized care based on “patient-journey” approaches compared to traditional maternal, newborn, and childcare programs like antenatal care, postnatal care, , and immunization. As emerging disease burdens such as noncommunicable diseases, including pediatric onset conditions (eg, sickle cell anemia, type 1 diabetes, congenital heart disease, mental health conditions, and developmental disorders), are becoming particularly important in South Asia, a combination of public health approaches and life-long individualized care is required. Countries are increasingly reorienting their health systems to a primary health care–centered approach with a strong community-based component, complementing traditional service delivery with essential public health functions, individual and community empowerment, and multisectoral action to advance health and well-being [1].

We envision a coherent digital and data architecture with a focus on longitudinal tracking for every child. Use of tools for early diagnosis and continuity of care across public and private health services and conditions and levels of service delivery (eg, community, primary health care facility, and specialized care) will help achieve rapid transformation in the health sector. Application of large AI and machine learning models on these longitudinal datasets can be a game-changer. Some of the use cases of effective application of AI and machine learning include sending targeted prevention or behavior change messages based on risk factors and self-diagnosis based on algorithms that direct high-risk clients to seek care. AI solutions can provide rapid diagnostics and clinical decision support for health care professionals and send reminders for compliance [24]. At the population level, such solutions can support supply chain optimization, better forecasting, process optimization, drug discovery, and further research and development.

Additionally, AI algorithms can help convert clinical records to standardized datasets (eg, International Classification of Diseases 10/11 or Systemized Nomenclature of Medicine–Clinical Terms). Rapid scale-up of edge technologies that capture data, like voice-based medical records, digital stethoscopes, and digital ultrasound machines, can further reduce the burden of data capture and support health workers to focus on client interaction and treatment. Large-scale design and implementation of such tools along with telemedicine, targeted messaging, e-prescriptions, and referral care hold tremendous potential to support health system functions to advance child health and well-being. Further, data models that predict, detect, and track emergencies and shocks with an impact on public health, such as severe climate events and emerging communicable diseases with pandemic potential, linked with early warning systems, public communication tools, and remote access to essential health care, are critical to enable health system readiness to respond to the impacts of climate change on child and adolescent health.

UNICEF in South Asia [25] has embarked on a journey to integrate the rapidly evolving frontier technologies into the next generation of digitally-enabled health systems, such as AI chatbots for child and adolescent mental health and community health worker support; data science for trend mapping and early detection of conditions ranging from outbreak-prone communicable diseases to neonatal defects; geographic information system mapping for microplanning and efficient service delivery; drones for vaccine deliveries; blockchain for logistics and supply chain management; and other emerging technologies. We are working closely with governments to design and introduce coherent digital health architectures that standardize and automate data processing across diverse and fragmented health information systems.

At the same time, UNICEF recognizes the entrenched digital divide in the South Asia region. We are partnering with global and regional agencies and the private sector to advocate for equitable digital access, address the network connectivity and affordability gap, and ensure that digital health services are contextually appropriate and accessible to everyone.

UNICEF in South Asia is committed to joining public and private sector partners in a united action to accelerate digitally enabled health systems, architecture planning, design and implementation of longitudinal tracking systems, and application of AI and machine learning models to improve the health and well-being of children, families, and communities across the region.

## Abbreviations

AI: artificial intelligence
UNICEF: United Nations Children's Fund

## Data Availability

The data underlying this article are available in the article and in its online supplementary material.
